# Sustainable data and metadata management at the BD2K-LINCS Data Coordination and Integration Center

**DOI:** 10.1038/sdata.2018.117

**Published:** 2018-06-19

**Authors:** Vasileios Stathias, Amar Koleti, Dušica Vidović, Daniel J. Cooper, Kathleen M. Jagodnik, Raymond Terryn, Michele Forlin, Caty Chung, Denis Torre, Nagi Ayad, Mario Medvedovic, Avi Ma'ayan, Ajay Pillai, Stephan C. Schürer

**Affiliations:** 1 BD2K-LINCS Data Coordination and Integration Center, University of Miami, Miami, FL 33136, USA; 2 Department of Human Genetics and Genomics, Miller School of Medicine, University of Miami, Miami, FL 33136, USA; 3 Department of Molecular and Cellular Pharmacology, Miller School of Medicine, University of Miami, Miami, FL 33136, USA; 4 Center for Computational Science, University of Miami, Miami, FL 33146, USA; 5 Department of Pharmacological Sciences, Icahn School of Medicine at Mount Sinai, New York, NY 10029, USA; 6 Department of Psychiatry and Behavioral Sciences, University of Miami, Miami, FL 33136, USA; 7 Division of Biostatistics and Bioinformatics, Department of Environmental Health, University of Cincinnati, Cincinnati, OH 45221, USA; 8 Division of Genome Sciences, National Human Genome Research Institute, National Institutes of Health, Bethesda, MD 20891, USA

**Keywords:** Systems biology, Computational biology and bioinformatics, Research data

## Abstract

The NIH-funded LINCS Consortium is creating an extensive reference library of cell-based perturbation response signatures and sophisticated informatics tools incorporating a large number of perturbagens, model systems, and assays. To date, more than 350 datasets have been generated including transcriptomics, proteomics, epigenomics, cell phenotype and competitive binding profiling assays. The large volume and variety of data necessitate rigorous data standards and effective data management including modular data processing pipelines and end-user interfaces to facilitate accurate and reliable data exchange, curation, validation, standardization, aggregation, integration, and end user access. Deep metadata annotations and the use of qualified data standards enable integration with many external resources. Here we describe the end-to-end data processing and management at the DCIC to generate a high-quality and persistent product. Our data management and stewardship solutions enable a functioning Consortium and make LINCS a valuable scientific resource that aligns with big data initiatives such as the BD2K NIH Program and concords with emerging data science best practices including the findable, accessible, interoperable, and reusable (FAIR) principles.

## Introduction

The Library of Integrated Network-based Cellular Signatures (LINCS) project^1^ is a multi-center NIH-funded program that is creating a comprehensive library of molecular signatures describing the effect of various perturbagens (e.g. small molecules, shRNAs, antibodies) on normal cellular functions, while also developing data integration, modeling and analysis methodologies. This extensive reference library of cellular responses is critical for understanding complex human diseases such as cancer, and could be further utilized to uncover new approaches for their treatment.

Large research consortia such as ENCODE^2^, TCGA^3^, HapMap^4^, and the 1000 Genomes Project^5^ have provided the research community with easily accessible resources that promote data reuse and data-driven scientific hypotheses that could subsequently be experimentally tested. The LINCS project employs a multitude of assays that span across a variety of technologies, models, systems, and readouts to collect data from many human cell types perturbed by a large number of diverse small molecules, genetic perturbations, and micro-environments. The six LINCS Data Signature Generation Centers (DSGCs) have employed more than 20 different assays in order to capture the perturbation effects on a variety of human cells via transcriptomics, proteomics, epigenomics, imaging, and ligand/competitive binding assays. More specifically:

1. The Broad Institute's LINCS Center for Transcriptomics (BroadT LINCS) is employing the L1000 assay^6^ to measure the effect of more than 25,000 chemical and genetic perturbations at the transcription level in over 50 human cell lines.
2. The Drug Toxicity Signature (DToxS) Generation Center at the Icahn School of Medicine at Mount Sinai is utilizing RNA-seq, Microwestern Proteomics, and Mass Spectrometry Proteomics assays to identify signatures related to the adverse events induced by FDA-approved drugs in the contexts of cardiotoxicity, hepatotoxicity and peripheral neuropathy.
3. The Harvard Medical School LINCS (HMS LINCS) Center is focusing on elucidating the response mechanisms of more than 100 cell lines and primary cells treated by about 400 small molecules and other ligands. HMS LINCS employs a wide variety of assays to characterize dose-response and time course effects on cell signaling and cell phenotypes upon diverse perturbations.
4. The Microenvironment Perturbagen LINCS (MEP LINCS) Center at the Oregon Health and Science University has developed a high-throughput imaging assay that measures the effect induced by microenvironment perturbagens on various biological processes such as proliferation, differentiation, and apoptosis. Overall, approximately 3,000 endogenous-ligand/microenvironment-perturbagen combinations have been studied in various cancer cell lines through the use of the Microenvironment Microarray (MEMA) assay^7^.
5. The NeuroLINCS Center at the University of California, Irvine is using the combination of high-throughput imaging, RNA-seq, ATAC-seq and SWATH-MS Proteomics to generate multifaceted cellular signatures of induced pluripotency stem cells (iPSCs) differentiated to motor neurons from amyotrophic lateral sclerosis (ALS) and spinal muscular atrophy (SMA) patient-derived cells.
6. The LINCS Proteomic Characterization Center for Signaling and Epigenetics (PCCSE) center at the Broad Institute is using the multiplex mass spectrometry-based P100 and GCP assays to generate a representation of the phosphoprotein and histone modification state of human cells before and after various chemical and genetic perturbations.

As of the 4th year of the Phase 2 of the LINCS Project, 22 assays are in production generating more than 350 datasets that contain almost 600,000 signatures and 1.37×10^7^ data points.

The BD2K-LINCS Data Coordination and Integration Center (DCIC) is tasked with processing and integrating these Big Data sets. This includes the registration and standardization of metadata, mapping to qualified external resources, and creating unique persistent identifiers. All LINCS data are openly available through a series of data releases on the LINCS Data Portal (http://lincsportal.ccs.miami.edu/), enabling scientists to address a broad range of basic research questions including the identification of prospective targets for therapeutic development, identification of cancer resistance mechanisms, prediction of possible mechanisms of action of drugs and other small molecules, assessing the repurposing potential of approved drugs, prioritizing synergistic drug combinations, as well as exploring the global space of all possible human cellular responses.

The BD2K-LINCS DCIC is also a part of the Big Data to Knowledge (BD2K) NIH Program^8^. Hence, the DCIC is developing and implementing general data science solutions involving data access and interoperability, persistent identifiers, data citation, and data reuse that could also be applied broadly to other projects. The BD2K-LINCS DCIC thus ensures that LINCS data are not only adequately processed and registered, but also that they are easily accessible and consistent with the findable, accessible, interoperable, and reusable (FAIR) principles^9^. As such, the data management practices and solutions at LINCS may serve as an exemplary format that can set the standard for future consortia and contribute to enhancing the transition to a more data-centric biomedical research environment. As biomedical screening technologies rapidly advance, with respect to both the resolution and the rate at which data can be generated, Big Data techniques in the acquisition, handling, and analysis of those data are increasingly important. Adopting rigorous Big Data management and processing practices has potential to improve many data-related issues that impede current research including reproducibility, data integration and reuse. Moreover, Big Data techniques can facilitate the use of data-driven research approaches in contrast to traditional case-based research approaches. One of the main challenges associated with this effort was the integration of the diverse datasets that have been produced by the DSGCs. The goal to integrate and model signatures generated by a variety of assay types that are in production at the DSGCs, motivated the DCIC to establish a unified and consistent platform to manage all those diverse datasets. In addition to the large diversity of data types, the DCIC had to account for the different operational informatics and management systems that were employed by the DSGCs and therefore had to establish flexible and customizable data processing workflows. Furthermore, these workflows had to adhere to the primary mission of the DCIC, which is ensuring the availability, accessibility, and persistence of the data within LINCS and the broader scientific and nonscientific communities. The solutions to the above challenges and the lessons learned can help to improve the data management practices in future Big Data and multi-center research consortia.

## Results

### LINCS Metadata Specifications

**Figure 1:**
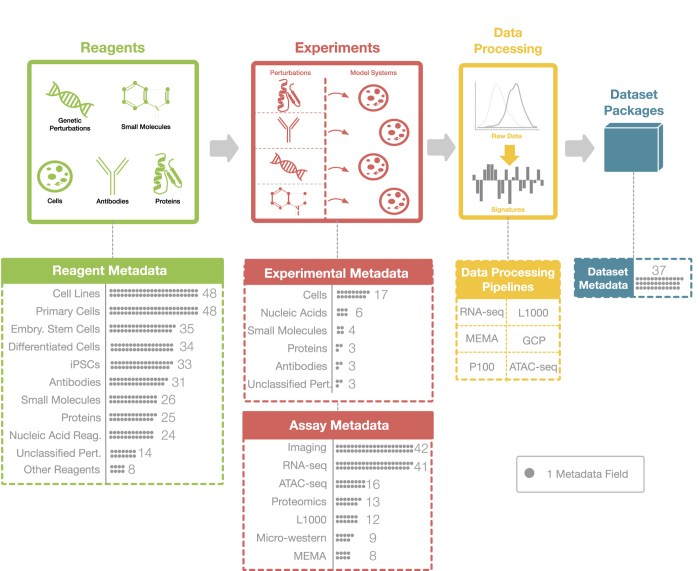
Overview of the LINCS Metadata including number of data fields for each category. The LINCS Metadata Specifications are divided into 4 categories. Reagent Metadata contain all the key information regarding the identity of 11 reagent types used in the LINCS experiments. Experimental Metadata fields describe the experimental conditions (e.g. concentration, time point) of those reagents. Assay Metadata describe all the reagent-independent experimental parameters. Dataset Metadata describe general information regarding attribution and properties of the datasets. Data (level) Processing Pipelines that generate the different data levels from raw data to signatures are currently available for 6 LINCS assays and are described including the software packages and version used, and key parameters/settings.

The size and diversity of the data produced by the LINCS Consortium necessitated the creation of extensive data standards specifications that would facilitate data exchange and integration within both the LINCS Project and the broader scientific community. For this, the DCIC, in close collaboration with the DSGCs, developed four categories of metadata specifications to capture essential attributes of the biological experiments, namely the critical reagents used (Reagent Metadata Specifications), experimental conditions (Experimental Metadata Specifications), reagent-independent assay parameters (Assay Metadata Specifications), and important dataset annotations (Dataset Metadata Specifications). These metadata categories are further divided into sub-categories and describe LINCS data generation, assays, reagents, and resulting datasets in detail (Fig. 1). The development of those standards followed the same process as in Phase I of the LINCS Project^10^: a Data Working Group (DWG) was formed with members from the DCIC and the DSGCs, and metadata use cases were created across all Centers. The LINCS metadata specifications can be found in Supplementary Tables 1, 2, 3, 4 as well as on the LINCS website (http://lincsproject.org/LINCS/data/standards) and for a complete list of acronyms used in this paper, refer to Table 1.

The purpose of the Reagent Metadata Specifications is to capture all the information necessary to identify the exact reagents used in any LINCS assay. Canonical fields, such as IDs for standardized small molecules, are critical for the integration of LINCS data across the Consortium and with external data sources. Batch fields provide important context and are important to evaluate the reproducibility of the LINCS experiments. To develop a comprehensive set of reagent metadata, the existing 6 metadata categories from Phase 1 (ref. 10) were expanded into a total of 11 categories. These 11 key categories (Small molecules, Cell lines, Primary cells, Embryonic stem cells, Differentiated cells, iPSCs, Nucleic acids reagents, Proteins, Antibody reagents, Unclassified perturbagens, and Other reagents) primarily include the perturbation-type model systems used in the LINCS assays, but also include reagents to detect analytes and quantified molecular changes (Fig. 1, Supp. Table 1). One major change compared with the previous version was the enhancement of the metadata fields using various minimum information standards^11^ (e.g. MIACA, MIAPAR, MIABE MIARE), to improve integration with external resources. All reagent categories share a set of common metadata fields (Common Fields), and each category also has unique metadata fields (Custom Fields). Moreover, all metadata fields were classified according to their importance (1=required, 2=recommended, 3=optional). Required descriptors (importance=1) are descriptors that are deemed most critical to identify, track, and describe reagents and must be provided by either the DSGCs or the DCIC (e.g. small molecule vendor name, vendor catalogue ID, vendor batch ID, etc.). For the recommended descriptors, the DSGC (and/or DCIC) is strongly encouraged to provide them as they contain details that inform of the biological context of the reagents (e.g. cell line donor age, sex, ethnicity, health status). Optional descriptors are less critical among the proposed specifications, but they still contain useful information to describe the reagent entities, and they should be provided if the information is available.

Particularly for the cell categories, the Reagent Metadata Specifications also capture the provenance of the cells (differentiated cells are derived from iPSCs, while iPSCs are derived from primary cells). Corresponding metadata are linked via the LINCS ID and the cell name (e.g. cells differentiated from iPCSs are linked to the corresponding iPSC metadata by capturing iPSC LINCS ID and the iPSC name). In this way, the derived cell inherits all metadata from its parent cell, and the relevant metadata are captured only once. All the Reagent Metadata Specifications can be found in Supplementary Table 1, are available on the LINCS website (http://lincsproject.org/LINCS/data/standards), and are also published as a community resource in FAIRsharing^12^.

The purpose of the Experimental Metadata Specifications is to capture the essential information that describe the experimental conditions linked to a LINCS reagent (e.g. small molecule concentration, cell density, etc.). That information plays an essential role for both the replication of an experiment as well as the interpretation of its results. For this, we created the appropriate experimental metadata fields for the following 6 categories: Small molecules, Cells (which include Cell lines, Primary cells, Differentiated cells, iPSCs, and Embryonic stem cells), Proteins, Antibody reagents, Nucleic acid reagents, and Unclassified perturbagens. Primarily, these standards are based upon existing standards such as Minimum Information About a Cellular Assay (MIACA)^13^ and Minimum Information About a Microarray Experiment (MIAME)^14^, as well as standards used by other consortia such as ENCODE. These Experimental Metadata Specifications were also used to build online, form-fillable templates in the Center for Expanded Data Annotation and Retrieval (CEDAR) system^15^, complete with controlled vocabulary responses through reference ontologies via the BioPortal^16^ when available. These templates can be found in Supplementary Table 2.

In addition to capturing information related to the reagents and the experimental conditions, it is also necessary to document all key assay parameters that were used in the LINCS experiments. Thus, the Assay Metadata Specifications were created for the most frequently used LINCS assays with the goal of providing details that are important, both for the replication of results and to evaluate the context in which data can be reused and integrated with similar assays from other sources (e.g. TCGA^3^, ENCODE^2^). As with the other metadata specifications, some fields were common across multiple assays (e.g. instruments, software version) while others were unique to the individual assay (e.g. read length in RNA-seq). LINCS assays were registered into the BioAssay Ontology (BAO)^17,18^ and cross-referenced and are also included in Supplementary Table 3.

Finally, a total of 37 non-experimental details including: Dataset Identifier, PI Name, Center Name, Grant Number, Dataset Release Date, QC Information, and more, that are associated with a scientific dataset, are captured by the Dataset Metadata Specifications (Fig. 1). These specifications provide a framework in which important dataset annotations for identification and attribution of the work are recorded in a unified way, enhancing the persistence of the datasets as they can easily be transferred to different dataset repositories. All the Dataset Metadata Specifications are available in Supplementary Table 4.

### LINCS Data Groups and Data Sets

**Figure 2:**
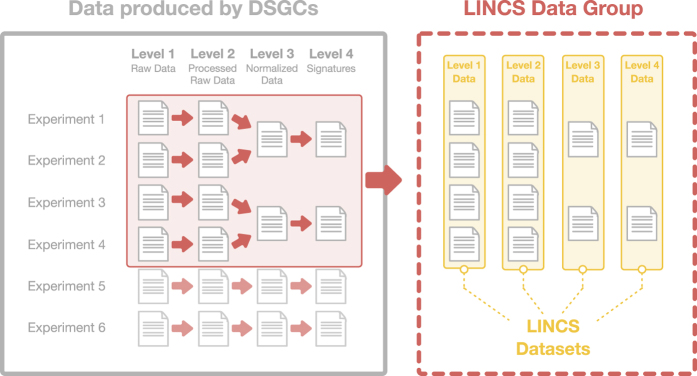
Organization of the LINCS Data Files. All data produced by the DSGCs are being organized into LINCS Data Groups and LINCS Datasets. A LINCS Data Group consists of a collection of experiments that contain all the generated data, regardless of their data level. Within a LINCS Data Group, the data files are further split into LINCS Datasets based on their data level.

All data produced by the DSGCs are split into distinct and assay-specific collections of experiments defined as LINCS Data Groups and are assigned a unique LINCS Data Group ID (LDG ID). The business rules for dividing the data into Data Groups follow each DSGC's suggestions and can be based on the time of the experiment (e.g. Data Groups from BroadT LINCS are partitioned based on the date of release of the data files) or the biological context of the experiment (e.g. Data Groups from PCCSE are partitioned based on the MOA class of the perturbagens used). The files within a LINCS Data Group are then organized according to their data levels and are assigned a unique LINCS Dataset ID for each data level that is made available (Fig. 2). The LINCS data levels are based on the work of the TCGA^3^ and have been developed according to a conceptual level of abstraction that is harmonized across all LINCS assays. They range from level 1 to 4 (or higher) where level 1 is the "raw data", level 2 processed data, level 3 normalized data that is useful for statistical analysis, and level 4 (or higher) is the aggregated signature level that can be used to connect different signatures. In principle, a dataset group can also contain multiple datasets of the same data level, for example different “flavors” of a signature. Links to the DSGCs data processing pipelines, via which raw data are transformed into the final signatures and their intermediate data levels, are included in the Dataset Metadata Specifications (Supplementary Table 4) under the field “Processing Pipeline”. Moreover, the pipeline parameters for many of the assays are included directly in the LINCS Dataset Packages in the “Processing Pipeline Specifications” file.

All LDG (LINCS Data Group) and LDS (LINCS DataSet) IDs that are assigned to the LINCS datasets are global and persistent enabling dataset citation (see below).

The DCIC makes accessible all data that meet the extensive quality control standards (QC) implemented by the DSGCs. This includes all data levels except the raw data (such as images or raw reads). Raw data including raw images and raw MS data are, at the current time, only accessible via the DSGCs. For several data types dedicated platforms exist or are being developed. Mass spectrometry raw data, for example, are available via Chorus (http://chorusproject.org), and image data are accessible via an Omero-based image server^19^. L1000 raw data are available via Gene Expression Omnibus (GEO)^20^. Those raw data are not duplicated by the DCIC, due to resource limitations to store and serve that data and, in some cases, proprietary data formats of the experimental instruments used. However, the DCIC facilitates the discovery of raw data by linking directly from the LINCS Data Portal.

Links to the QC standards and SOPs used by the DSGCs are recorded in the Dataset Metadata Specifications of each LINCS Dataset under the field “Dataset QC/QA information”.

### Overview of the Data Processing Pipeline

**Figure 3:**
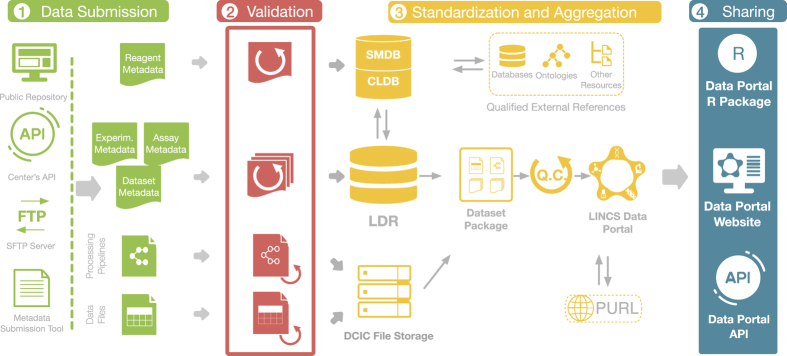
Overview of the Data Processing Pipeline. The Data Processing Pipeline consists of four main steps. Data Submission: data and metadata generated by the DSCGs are transferred to the DCIC via one of several technological solutions. Validation: the format and terminology of the data and metadata get validated according to the internal and qualified external references. Standardization and Aggregation: Submitted Reagent Metadata are standardized and further validated and annotated using qualified external references. Small Molecules and Cell Lines are registered into dedicated registration systems (SMDB, Small Molecule DataBase and CLDB, Cell Line DataBase) and are also assigned global IDs (PURLs). The Experimental, Dataset and Assay Metadata are directly deposited into the LDR. Processing pipelines and data files are deposited into the DCIC File Storage. The LDR and the DCIC File Storage are then used for the creation of the LINCS Dataset Packages. After quality control, released Dataset Packages are assigned global PURLs and made accessible via the LDP. Data Packages can be accessed via the LDP UI, through APIs and the LDP R package. Arrows indicate the flow of information between the four main processing steps.

An important task of the DCIC is to make accessible to the scientific community the data produced by the DSGCs in a harmonized, streamlined format including deep metadata. For this reason, the DCIC has developed a robust processing pipeline that involves various validation and quality control (QC) steps, data standardization, identification, aggregation, cross-referencing, and dataset packaging to make LINCS data and metadata accessible in a consistent and harmonized format that facilitates integration (Fig. 3). Overall, the processing of the LINCS data and metadata can be divided into 4 interconnected steps (Fig. 3) and are described in detail in the following sections.

Briefly, the data and associated metadata are first obtained from the DSGCs, and after being vetted for quality (see Validation below), they are recorded in the LINCS Data Registry (LDR), a database that captures associations of all metadata, assays, and datasets. After standardization, unique IDs are generated, metadata are cross-referenced and enriched by various external resources, and data are aggregated/combined and packaged into consumable standardized and harmonized dataset packages.

### Data Submission

Accurate, fast, and effortless exchange of information between the DSGCs and the DCIC is essential for the success of the Project. Since there are multiple DSGCs, the DCIC had to accommodate for the uniqueness of each Center's operations and informatics infrastructure. Therefore, the DCIC has employed multiple ways to connect with the DSGCs that ensure an unobstructed and compatible flow of information. More specifically, original data files were transferred from the DSGCs to the DCIC by using either: (1) DCIC’s SFTP service, (2) download from public data repositories such as GEO^20^ or Synapse (https://www.synapse.org/) or (3) APIs or direct download from the DSGC’s website (Fig. 3).

Similarly, metadata collection was also performed in multiple ways, considering each Center's specific operational infrastructure. To ensure adherence to the LINCS Metadata Specifications, a Metadata Submission System (http://lincsportal.ccs.miami.edu/mst/) was developed and implemented. Through this online tool, the Centers can readily submit their reagent metadata and map their Center-specific naming conventions to the official LINCS Metadata Specifications terminology. As an alternative to this GUI-based software system, DSGCs can also submit their metadata using APIs from their in-house databases. For this, proper mapping between a Center's APIs terminology and the approved LINCS Metadata Specifications was established to ensure the accurate transfer of the metadata.

In addition to data and metadata, the DCIC also obtains the data-level processing pipelines from the DSGCs, typically via a GitHub repository.

**Figure 4:**
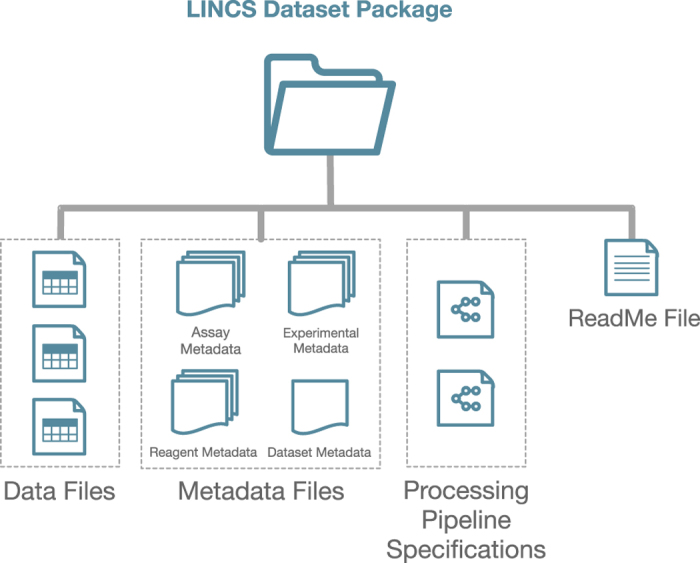
Components of a LINCS Dataset Package. Each LINCS Dataset Package consists of the Data Files that were produced by the DSGCs, the Reagent Metadata and Experimental Metadata for each reagent used in the experiment, the Assay Metadata describing the assay used in the experiment, the Dataset Metadata describing the dataset, the Processing Pipeline Specifications that contain parameters used for the processing of the data and the ReadMe file that contains the manifest of the Dataset Package.

Once the DSGC data and metadata files are received by the DCIC, they enter an extensive modular data processing pipeline that is described in the following sections and that briefly includes: data type and content validation; standardization of metadata records; registration of reagents, and generation of unique IDs; addition of annotations from external resources; aggregation of all the data, and dataset packaging; QC, and final approval by the DSGCs; and publication. This process results in clean, standardized dataset packages (Fig. 4) that are consistent across assays and sources with harmonized annotations and IDs to ensure that LINCS datasets from all Centers are compatible and can be integrated and analyzed.

### Validation

First, all data, metadata, and processing pipelines pass through a validation step. Metadata entries are validated based on the data file format; completeness of the required fields and content, e.g. the terminology artifact for the content of a field; and also, the content itself via manual review and cross-referencing where possible. Fields in a submitted data file are mapped to LINCS data fields, and the submitted content is compared to registered LINCS reagents to identify potential errors, and to ensure that referenced metadata has been previously registered and that all required data fields (annotations) are available. The formal data submission requires that new reagents have been previously registered into the system. The DCIC also validates the data processing pipelines employed by the DSGCs to transform their raw results into the final signatures, typically via several steps that each generate a data level (from 1 to 4; see Supplementary Table 4).

### Standardization and Aggregation

After initial validation, all data files and metadata enter the Standardization and Aggregation process. Here, the dataset files are assigned their respective LINCS Dataset (LDS) ID and are grouped into their respective LINCS Data Group (LDG) ID and are uploaded to the DCIC file storage so that they are easily accessible. The experimental, assay, and dataset metadata files are submitted to the LINCS Data Registry (LDR). Submitted reagent metadata proceed into the standardization pipeline. The purpose of standardization is to generate a canonical representation for each key reagent (such as small molecule, cell, protein, etc.), based on which a unique LINCS ID is assigned. Reagents that are already known to the system (previously registered) are cross-referenced to their existing (canonical) record (by LINCS ID). For new reagents, new LINCS IDs are created after standardization following the DCIC business rules. For example, small molecules used in LINCS experiments undergo the standardization process and get assigned a new or already existing LINCS ID based on their canonicalized unique chemical structure representations. The LINCS Data Registry (LDR) system then keeps track of all submitted samples (or batches) and the specific datasets in which they are used (based on the submitted sample-specific batch ID), and tracks the associated standardized canonical LINCS ID to enable data integration. The standardization process is required to enable integration of datasets associated with the same reagents (e.g. small molecules or cells), but that were generated by different DSGCs and therefore use different physical samples, from different suppliers with potentially varying representations or names. Standardization is also required for integration with external resources (see below). Once all key reagents of the dataset are registered in the LDR, all data and metadata are packaged. A LINCS Dataset Package (Fig. 4) is a container file that is consistent and uniform across different data types and different Centers. It includes: (i) a ReadMe file that contains the package manifest; (ii) the original Data Files as submitted by the DSGCs; (iii) Reagent Metadata files for all the reagent categories that were used in the experiment including all fields and canonical LINCS IDs; (iv) Assay and Experimental Metadata Files that describe the conditions and parameters of the experiment that corresponds to each reagent category and to the assay in general according to the specifications; (v) Dataset Metadata Files that contain standardized information regarding the dataset such as the DSGC Name, PI Name, Assay Name, Grant Number, Released Date, and links to the Processing Pipelines; and (vi) Processing Pipeline Specifications that contain the parameters that were used for the processing of the data.

To enable dataset citation of the LINCS data, we followed the Joint Declaration of Data Citation Principles (JDDCP)^21^. We registered collections that correspond to categories of LINCS digital research objects in the Minimal Information Required in the Annotation of Models (MIRIAM) Registry to generate unique, perennial, and location-independent identifiers^22^. These collections include data level-specific dataset packages and dataset groups (for all data levels). The identifiers.org service, which is built on top of the information stored in MIRIAM, provides directly resolvable identifiers in the form of Uniform Resource Locators (URLs). This system thus provides a globally unique identification scheme to which any external resource can point and a resolution system that gives the owner/creator of the resource collection flexibility to update the resolving URL without changing the global identifiers. LINCS identifiers currently resolve to the LINCS Data Portal (LDP) Dataset landing pages (http://lincsportal.ccs.miami.edu/datasets/). These dataset and dataset group identifiers are the central component of the LINCS dataset citation record, which further includes the authors, title, year, repository, resource type, and version, which are also available via the LDP. URIs (Uniform Resource Identifiers) for LINCS datasets are one of the required component of the FAIR principles^9^.

### External Data Sources Mapping, Curation, and Annotation

**Figure 5:**
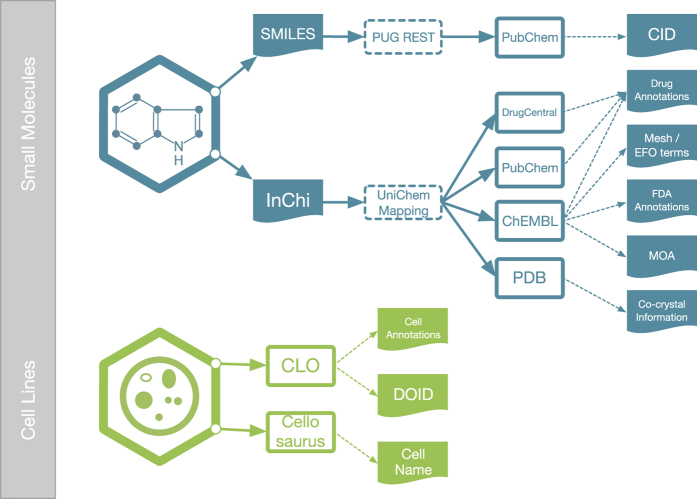
Qualified External Resources for the Small Molecule and Cell Line Annotations. Small Molecules used in the LINCS assays are annotated using PubChem, DrugCentral, ChEMBL and PDB; mappings are made via Web Services that are based on SMILES and InChIs. LINCS Cell Lines are mapped and further annotated by CLO and Cellosaurus.

An important step in the data processing pipeline is the enhancement and validation of the Center-submitted metadata by the use of qualified external references. For the Small Molecule metadata sub-category, external resources are used to validate the chemical structure representation and identity, and to provide additional annotations, such as mechanism of action targets, disease associations, clinical phase status, synonymous names, etc. (Fig. 5). The enriched and validated annotations are then deposited into the SMDB (Small Molecule Database), a database created specifically for hosting small molecule information (Fig. 3). LINCS small molecule structures are standardized via a largely automated pipeline including removal of predefined salt and addend forms, neutralizing charges of deprotonated acid and protonated base forms, and generating a unique chemically reasonable tautomeric representation and SMILES canonicalization. This pipeline has several checkpoints to detect exceptions. Because it is not practical to handle all possible exceptions in a completely automated process, these are manually reviewed and curated and then added back into the pipeline. The standardized structures are then mapped to PubChem compound identifiers (CIDs) via structure-based query of PubChem's Power User Gateway API (PUG REST, https://pubchemdocs.ncbi.nlm.nih.gov/pug-rest). CIDs submitted by DSGCs are compared and conflicts resolved. Additional validation steps include the PubChem standardization service and resolution of any conflicts that arise after applying the DCIC standardization pipeline. Subsequent queries to PUG REST, via mapped CIDs, also return chemical probe information for LINCS small molecules. LINCS small molecule structures are further cross-referenced to all major public chemical databases via the UniChem^23^ mapping service that is based on InChI (IUPAC International Chemical Identifier)^24^ representations. Via this mapping, identifiers for LINCS compounds from ChEBI^25^, ChEMBL^26^, PubChem^27^, DrugCentral^28^, Protein Data Bank (PDB)^29^, and BindingDB^30^ were incorporated. The ChEMBL database (version 22 for the currently released LINCS datasets) was further used to extract target-specific mechanism of action information; MeSH (Medical Subject Headings)^31^ and Experimental Factor Ontology (EFO)^32^ disease indication terms; and Federal Drug Administration (FDA) approval annotations for LINCS compounds. Cross-references to PDB co-crystal protein structures of LINCS compounds and additional DrugBank and BindingDB target and disease annotations will be added in the near future via custom pipelines leveraging the respective APIs to corroborate and extend current ChEMBL bioactivity and drug annotations. Target-specific bioactivity annotations for LINCS compounds were curated from the ChEMBL database after filtering by various criteria including confidence score≥5; assay type (e.g. binding or functional); standard result type (endpoint) Potency, IC_50_, K_d_, K_i_, Activity, EC_50_, and AC_50_ of only concentration-response data. Compound activity values were p- (-log10) transformed; grouped by LSM ID (LINCS Small Molecule ID), ChEMBL target ID, and result type; and averaged to obtain an aggregated activity value for each available compound/target/endpoint combination. For each aggregated value, the target protein was assigned a standard gene symbol/name identifier by querying the UniProt database^33^ with the accession number provided in the ChEMBL record.

In addition to the Small Molecules, Cell Line Metadata were also integrated with various external sources (Fig. 5). First, Cell Line Metadata were mapped to and integrated with the Cell Line Ontology (CLO)^34^. To harmonize LINCS cell line representation in the CLO, CLO design patterns were slightly updated to add new information of the LINCS cell lines including different database cross-reference IDs^35^. Moreover, a new shortcut relation was generated to directly link a cell line to the disease of the patient from whom the cell line was originated. Disease annotations for cell lines were curated and mapped to the disease ontology (DO)^36^; these curated annotations were also consolidated with DO annotations in CLO. In addition, cell lines were cross-referenced with Cellosaurus (http://web.expasy.org/cellosaurus/).

### Data Release and Access

LINCS data and metadata management business rules, processes, and infrastructure described above provide the backbone of the LINCS Data Portal (LDP; http://lincsportal.ccs.miami.edu/), an advanced search engine that facilitates querying, filtering, exploration, and downloading of all LINCS datasets, metadata, and related curated content based on numerous categories and functions^37^.

The LINCS Data Portal is organized by major content categories, such as small molecules, cells, and, most importantly, datasets. In LDP, each LINCS Data Group has its own landing page. Within the landing page, a user can access the experimental data files and their corresponding reagent, experimental, assay, and dataset metadata and their curated annotations. Via various user interface functionalities, LDP facilitates exploring LINCS data, enabling browsing through the various LINCS assays and select different LINCS datasets including those generated in the LINCS Pilot Phase (Phase 1).

In addition, users can also access the LINCS Data Portal programmatically through a RESTful API (http://lincsportal.ccs.miami.edu/apis/) and the R package LINCSDataPortal (https://github.com/schurerlab/LINCSDataPortal/). This package provides easy access to all the data and metadata that are stored in the LINCS Data Registry. More specifically, LINCS data packages can be retrieved using a variety of search terms for entities of interest (e.g. small molecule, protein, gene, cell line) and downloaded for any of the available data levels. Metadata associated with any reagent used in LINCS experiments can also be retrieved. Having developed these tools, the DCIC makes LINCS content conveniently accessible to computational researchers as well as wet-lab experimentalists.

### Available LINCS data and FAIRness

**Figure 6:**
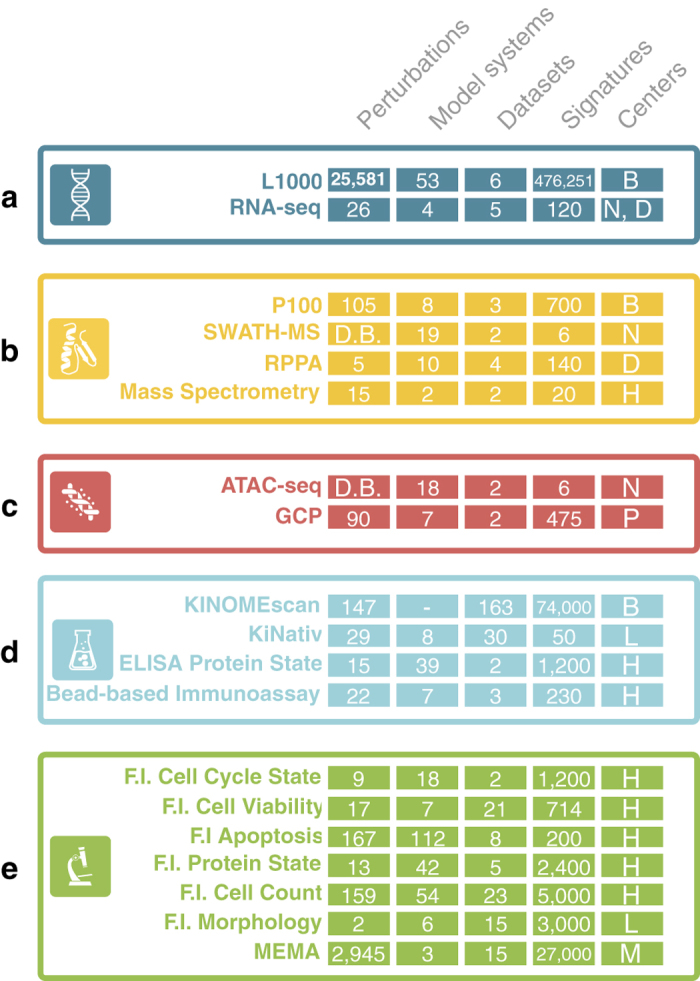
Overview of the LINCS Assays by Subject Area. LINCS Assays are classified according to a generic subject area (**a**: transcriptomics, **b**: proteomics, **c**: epigenomics, **d**: binding, **e**: imaging). For each assay, the number of unique perturbations, model systems, datasets, profiles (signatures) and the DSGC is annotated. Only assays used by DSGCs of LINCS Phase 2 are shown. Perturbations include small molecules, genetic antibodies, and other ligands. Model systems include cell lines, primary cells, differentiated cells, iPSCs and Embryonic Stem Cells. **b**: BroadT LINCS, P: PCCSE, N: NeuroLINCS, H: HMS LINCS, D: DtoxS, M: MEP LINCS, D.B.: Disease Background, F.I.: Fluorescence Imaging.

To date, the LINCS project has published over 350 datasets with almost 42,000 small molecule perturbagens, and over 1000 cells types. Figure 6 illustrates the perturbagens (including small molecules, genetic perturbations such as gene knockdown, and microenvironments), model systems (such as cell lines, iPCS, and primarily cells); and the number of datasets and derived signatures and Centers by 'Omics' category including transcriptomics, proteomics, epigenomics, protein binding, and imaging (cellular phenomics) with several subcategories describing the phenotype. All datasets, metadata, processing pipelines, and rich annotations are available via the LINCS Data Portal (LDP, http://lincsportal.ccs.miami.edu/dcic-portal/).

The FAIR Guiding Principles^9^ seek to enhance the ability to find, access, integrate/interoperate with, and reuse datasets. The LINCS DCIC is actively involved in BD2K and NIH Commons initiatives including development and evaluation of data FAIRness^38^. LINCS datasets and metadata reagents are indexed and mapped by cross-domain-interest initiatives such as DataMed^39^, OMICS Discovery Index^40^, ChEMBL^26^, ChEBI^25^, CLO^34^, and Cellosaurus (http://web.expasy.org/cellosaurus/). Creating standardized perennial IDs for LINCS digital research objects and extensive metadata annotations, along with the use of qualified community terminology artifacts and ontologies, cross-references and indexing, dataset citation, and data access via several mechanisms including well-defined APIs, all contribute to make LINCS data FAIR.

## Discussion

Large-scale multi-omics, phenotypic profiling, and drug screening technologies have the potential to revolutionize biomedical research and drug discovery by enabling systems-wide modeling of the complex and dynamic networks of molecules and biological processes involved in human disease.

Such large-scale disease modeling efforts not only require the generation of large volumes and diverse types of data, but also necessitate the implementation of scalable data management, integration and analysis approaches. The LINCS project is building an extensive diverse reference library of cell-based perturbation-response signatures, along with novel data analytics tools to improve our understanding of human diseases at the systems level. LINCS employs a wide range of assay technologies, perturbations, cell model systems and cellular readouts (Fig. 6). Data management including harmonization across the various data types, standardization, aggregation, integration, and analytics are therefore particularly challenging. Specifically, the dataset packages assembled by the DCIC and accompanying metadata need to support Big Data approaches by enabling ready integration of the LINCS signatures and mapping to external resources via rigorous standardization of perturbations, model systems, and results, while also providing in-depth experimental and assay annotations required to understand and analyze the rich biological results datasets. In addition, it is important that LINCS data products can be easily located by researchers, for example via catalogs such as DataMed or OmicsDI, and accessed via a variety of protocols and tools such as APIs, the LINCS Data Portal UI, an R package and other tools such as the iLINCS platform (http://www.ilincs.org/ilincs/), L1000FWD^41^, Enrichr^42^, SEP-L1000^43^, and the L1000CDS^2^ search tool^44^. Deep metadata annotations based on community reporting standards and the use of established terminologies and ontologies contribute to interoperability and reusability of the LINCS data. Datasets must be citable to attribute them to the DSGCs and to reference specific datasets in any products that use LINCS data.

In addition to data and metadata processing under the control of the DCIC (as described above), effective data management requires efficient communication and information exchange between the data producers (the DSGCs) and the DCIC. This interface has to accommodate each Center's internal operations and corresponding operational informatics infrastructure, which determines, for example, reagent registration, dataset and metadata management, and the submission of data to the DCIC. In practice, there are significant differences in informatics resources and capabilities. Modularization of the data processing pipeline to accommodate different, and in some cases customized, technical solutions for data exchange and submission, is therefore particularly important. In addition, the DCIC has to provide technical support via open communication channels and expertise as needed to resolve any deficiencies. The LINCS Metadata Specifications represent a critical common "reference" for all Centers as the foundation for internal and external data integration and validation.

Our data management and stewardship solutions contribute to a functioning Consortium and to make LINCS a valuable scientific resource that concords with emerging data science best practices including the FAIR principles and with far-reaching long-term scientific impact.

## Methods

### Metadata Specifications

LINCS Metadata Specifications (Reagent, Experimental, Assay and Dataset) were developed following the same process previously described^10^. An important mechanism to review, improve, and approve draft specifications was the LINCS Data Working Group (DWG) that meets monthly with representatives from the LINCS DSGCs, DCIC, and NIH.

### Mapping to Data Standards and Ontologies

The fields to be captured in the respective metadata standards categories were informed by community reporting guidelines including Minimum Information About a Cellular Assay (MIACA), available via FAIRsharing, and efforts in other research Consortia such as ENCODE. Similarly, established controlled terminology artifacts were identified and incorporated as indicated in the metadata standards, including terms related to taxonomy, disease, identity, and function. Linkages to external resources and repositories include field name and parameter matching and utilization of ontological branches to enable controlled vocabulary for metadata field names and values, such as mapping to the Cell Line Ontology (CLO) and ChEMBL data repositories (see External Data Annotation section).

### Small Molecule Standardization and External Resource Annotation

To enable exact compound identification and therefore facilitate LINCS data integration, we developed a robust registration pipeline for small molecules. Chemical structures are standardized via a series of rules, including removal of salt and added forms, charge neutralization (deprotonated acids and protonated bases), and generation of a unique and stable tautomer forms. These rules have been developed and optimized to consolidate chemically identical small molecule representations which exhibit variations in their submitted structural representations. Standardization protocols are implemented using BIOVIA Pipeline Pilot v.16.2 ((http://accelrys.com/products/collaborative-science/biovia-pipeline-pilot/) and ChemAxon Standardizer v17 (https://chemaxon.com/) tools, both of which are widely used chemical/pharmacological informatics technologies. Post-standardization, the registration process involves comparison and mapping of standardized molecules to many external chemical structure databases. Additional external data are currently extracted from ChEMBL and PubChem (other resources such as DrugBank and BindingDB will be incorporated soon) and mapped to the LINCS compounds via custom pipelines implemented in Pipeline Pilot, Python, and PostgreSQL. The endpoint of small molecule registration is a dedicated small molecule database (SMDB) which streamlines input, output, and data storage for the stages of the registration pipeline and provides accessibility and usability of externally mapped information. SMDB also captures provenance of chemical structures and samples and their annotations from external reference resources.

### Data Processing Pipeline

Modular data processing pipelines were built using BIOVIA Pipeline Pilot v16.2 (http://accelrys.com/products/collaborative-science/biovia-pipeline-pilot/). Various components and scripts were designed to parse data and metadata from databases, via APIs, and data files located in the SFTP server. The collection of pipelines performs field mappings, data joining and merging, basic validation and contains several checkpoints for manual data review. Prior to processing through Pipeline Pilot protocols, preprocessing steps are performed for all gct and gctx files (generated by BroadT LINCS and PCCSE) through the parse.gctx function of the cmapR R package (https://github.com/cmap/cmapR).

### LINCS Data Registry

The LINCS Data Registry (LDR) is the main data management system for the LINCS DCIC and serves as the primary data source for the LDP (LINCS Data Portal), LINCSDataPortal R package and the RESTful API. LDR combines a key-value store with a traditional relational database schema. The key-value store captures all metadata fields and the relational schema captures the associations between centers and their associated assays, datasets reagents, and their corresponding metadata. This system provides flexibility to implement changes as the metadata specifications evolve while it ensures data integrity across the multiple datasets and their associated reagents.

### LINCS Data Portal and RESTful API

All the LINCS data and metadata can be accessed through the LINCS Data Portal (http://lincsportal.ccs.miami.edu/), the LINCSdataportal R package (https://github.com/schurerlab/LINCSDataPortal) and a RESTful API. The LDP is a Web-based application that used PostgreSQL and Apache Solr for data storage, Angular Javascript (JS) for presentation, and Java-based servlets deployed on Tomcat for the service layer. The RESTful API utilizes the Solr index to fetch the requested data and make them programmatically available.

## Additional information

**How to cite this article**: Stathias, V. *et al*. Sustainable data and metadata management at the BD2K-LINCS Data Coordination and Integration Center. *Sci. Data* 5:180117 doi: 10.1038/sdata.2018.117 (2018).

**Publisher’s note**: Springer Nature remains neutral with regard to jurisdictional claims in published maps and institutional affiliations.

## Supplementary Material

Supplementary Table 1

Supplementary Table 2

Supplementary Table 3

Supplementary Table 4

## Figures and Tables

**Table 1:** Definitions of common acronyms.

Acronym	Definition	Description
BD2K	Big Data to Knowledge Program	An NIH Common Fund Program
DCIC	Data Coordination and Integration Center	Dedicated Center for processing and integrating the LINCS Data
DSGCs	Data Signature Generation Centers	6 Centers that generate the LINCS data by using more than 20 assays
DWG	Data Working Group	A LINCS working group with the goal of reviewing and improving specifications
FAIR principles	Findable, Accessible, Interoperable, and Reusable principles	Guiding principles for facilitating the reusue of scholarly data
LDP	LINCS Data Portal	The primary web/data portal for accessing the LINCS Data
LDR	LINCS Data Registry	The main management system for the LINCS DCIC
LINCS	Library of Integrated Network-based Cellular Signatures	An NIH Common Fund program with the goal to establish a network-based understanding of biology
